# Evaluating the effectiveness of personal resilience and enrichment programme (PREP) for HIV prevention among female sex workers: a randomised controlled trial

**DOI:** 10.1186/1471-2458-13-683

**Published:** 2013-07-26

**Authors:** Winnie Wing-Yan Yuen, William Chi-Wai Wong, Catherine So-Kum Tang, Eleanor Holroyd, Agnes Fung-Yee Tiwari, Daniel Yee-Tak Fong, Weng Yee Chin

**Affiliations:** 1 Department of Family Medicine & Primary Care, 3/F., Ap Lei Chau Clinic, The University of Hong Kong, 161 Main Street, Ap Lei Chau, Hong Kong, China; 2 Department of Psychology, Faculty of Arts and Social Sciences, National University of Singapore, Block AS4, #02-07, 9 Arts Link, Kint Ridge 117570, Singapore; 3 Asian and Gender Studies, RMIT University, PO Box 71, Bundoora 30038, Australia; 4 School of Nursing, Li Ka Shing Faculty of Medicine, The University of Hong Kong, Hong Kong, China

**Keywords:** Randomised controlled trial, Female sex workers, HIV prevention, Resilience, Protocol

## Abstract

**Background:**

Female sex workers (FSWs) are often considered as the vector, if not reservoir, of HIV and other sexually transmitted infections. Building upon the existing evidence on the role of psychological health in sexual health, the aim of this protocol is to describe a trial investigating the effectiveness of the Personal Resilience and Enrichment Programme (PREP), a resilience-promoting intervention that targets at psychological well-being i.e. self-esteem, self-efficacy and coping, to facilitate adaptation and ultimately safe sexual practices among FSWs, which could be an innovative strategy in controlling the spread of these infections.

**Methods:**

A total of 132 FSWs will be recruited and randomly assigned to either the intervention or usual care (control) groups in a multi-centred randomised controlled trial. Based on the resilience framework, this intervention is comprised of six weekly sessions focused on the awareness, expression and management of emotions, identifying roles and personal strengths, and effective problem-solving skills. Complex intervention assessment on both intervention process and effectiveness will be adopted when the primary outcome reduction of sexual risk behaviour and other psychological outcomes include their perceived stress, self-esteem, self-efficacy, coping overall resilience, and psychological distress will be measured at baseline, post-treatment and 3-month post-intervention and differences assessed by ANOVA. The relationship of resilience factors, psychological health and HIV preventive behaviours will be evaluated using structural equation modelling.

**Discussion:**

It is anticipated that this study will increase our understanding of the relationships between individual resilience attributes, positive adaptation, psychological health and sexual health practices. If successful, this programme will provide an innovative direction for HIV prevention by applying the personal resilience factors to promote both psychological well-being and safe sex for this high risk population.

**Trial Registration:**

ChiCTR-PRC-13003091

## Background

HIV infection remains a global public health challenge. In low-to-middle income countries in Eastern Europe and Asia, there was a 25% increase in new infections between 2001–09 [1]. In order to reduce transmission and the spread of infections, targeted programmes at high-risk populations are essential [2]. Among various high-risk groups, female sex workers (FSWs), who are associated with a greater number of sexual partners compounded by high mobility, have been identified to play a significant role in the transmission of HIV and other sexually transmitted infections (STIs) [3]. This is due to the fact that FSW could potentially act as a “bridge” to spread the virus to their clients and families of their clients and so forth to the general public, and to the neighbouring regions and vice versa. According to the Global Health Sector Strategy on HIV/AID 2011–15 recently published by the World Health Organisation (WHO), identifying and optimising contextually-tailored HIV prevention programmes for high risks populations was strongly recommended [4].

Current HIV prevention strategies for FSWs include harm reduction, biomedical and structural interventions operating at individual and population levels. Systematic reviews on the effectiveness of these HIV prevention programmes indicated that harm reduction programmes based on the Health Belief Model [5], Theory of Reasoned Action [6], or Theory of Planned Behaviour [7] are commonly used and mainly involve promoting condom use by increasing associated knowledge, removing barriers for access, or changing their beliefs and attitudes. In addition, interventions targeted at FSWs are effective [8,9]. Nonetheless, these programmes seldom address the issues that initially bring these women into commercial sex and the associated psychological distress. It is thus argued that since many of these interventions do not take into consideration the emotion and personal circumstances of the target population, they are unlikely to have sustainable effects [10].

Indeed, there is strong evidence of poor psychological health among FSWs associated with sex work, such as lower quality of life, higher likelihood of self-harm, anxiety and depression [11-13]. The long waiting hours for business, risks of being verbal abused, physically attacked, raped or even robbed by clients, along with the stigma attached to sex work and other related personal life circumstances [14] are together highly stressful. A number of studies have reported that FSWs with psychological distress were more likely to engage in HIV related risk behaviours such as substance abuse and inconsistent condom use [15-17]. Therefore, developing effective interventions to reduce their psychological distress and improve psychological well-being could have considerable potential in informing future directions for HIV prevention.

### Resilience as a new direction for HIV prevention

When individuals are exposed to traumatic or stressful situations, some may display psychological distress while others may have the ability to positively adapt to adversity and function competently. Such positive adjustments are referred to as *resilience*[18-20]. Based on the personal, familial, and environmental factors identified in previous studies, several prevention interventions have been proposed and demonstrated their effectiveness amongst young people; for example, the Bounce Back program [21], the Bright Ideas [22] for promoting resilience and coping skills, the Penn Resiliency Program [23] for promoting optimism and preventing depression, and the Stress Inoculation Training [24,25] for preventing anxiety amongst adolescents.

Amongst the few controlled studies investigating resilience training in adults, several have significantly improved participants’ psychological well-being and lowered distress. For example, the Well-being Therapy [26], based on Ryff’s multidimensional model of psychology well-being [27] including autonomy, personal growth, environmental mastery, purpose in life, positive relations and self-acceptance, was delivered in 4-to-8 sessions every other week and found to be effective in enhancing psychological well-being in adolescents and adults when tested alone or with cognitive-behavioural techniques [28,29]. Another resilience programme, the Transforming Lives through Resilience Education [30] which comprised four two-hour sessions, has shown to promote resilience and coping strategies through psycho-education, cognitive strategies, social skills training in college students.

Although the aforementioned studies have shown long-term improvements in psychological outcomes, there are potential difficulties in directly applying these resilience-promotion programmes to a marginalised group of women such as FSWs, who face specific but different sets of stressors and life circumstances from women who are not sex workers. With that aim in mind, our team has conducted a qualitative study on 23 FSWs in Hong Kong in 2012 [31]. FSWs interviewed in this study reported perceived and public stigma to the extent that they would not disclose their occupation and tended to reduce contact with their friends, partners or family which implied a paucity of social or family support and resources that could be drawn upon. As a result, unitising resources that lie within an individual would be a more important and acceptable approach to enhance positive adjustment in this population.

Researchers have identified a number of resilience factors which enable an individual to function competently in stressful situations. Individuals with high self-esteem were more likely to engage in health-promoting behaviours, such as using condoms or other contraceptives regularly [32] whereas people with low self-esteem and negative mood reported an increased propensity to engage in unprotected sex [33]. In addition to self-esteem, self-efficacy which refers to an individual’s belief in their ability to achieve desired goals [34], is another resilient factor that protects individuals against minor and major stresses [35,36], and promotes physical well-being. The Transactional Model of Stress and Coping [37,38] proposes that when a person faces stressful events, he or she would evaluate the situation to determine which types of coping strategies they would engage in [39]. Results from two large scale surveys in USA [40,41] investigating life stress, coping and psychological distress suggested that people who had used more coping strategies in response to stressors reported lower levels of emotional distress. This was also illustrated in our qualitative study in that FSWs who possessed personal strengths of greater self-esteem, stronger feeling of self-efficacy appeared to be associated with a reduced risk of psychological problem, lower sexual risk behaviours and better psychological well-being. Thus, the active engagement of positive psychology and resilience that strengthen and promote personal resources found within FSWs may provide a new direction for remediating stress, promoting self-esteem and thus better self-care and safe sex.

#### Research aims and objectives

The Personal Resilience and Enrichment Programme (PREP) has been developed according to resilience framework for a trial with FSWs in Hong Kong. The aim of this study is to seek a new direction for HIV prevention by investigating the effectiveness of PREP in FSWs. Through promoting FSW psychological health, it is hypothesised FSWs will take better care of their physical well-being and actively practise HIV preventive measures, and hence reduce their HIV risks. Specifically, the objectives of this study include:

1. To evaluate the effectiveness of PREP, the resilience-promoting intervention, in enhancing FSWs’ overall resilience, reducing their psychological distress, and eventually reducing HIV risk behaviours among FSWs in Hong Kong.

2. To explore the mechanism of how individual resilience factors operate to promote better psychological outcomes and decreasing HIV infection among FSWs.

**Figure 1:**
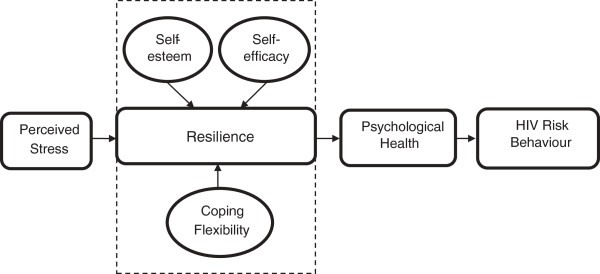
Proposed model of individual resources in predicting HIV risk behaviours.

This study is a randomised controlled trial to test the hypothesis that the participants in intervention group, as compared to those in the control group receiving usual care, would have an improvement in HIV preventive behaviour through the increase in overall resilience and in terms of their self-efficacy, self-esteem and coping strategies. At the same time, it is hypothesised that this cohort of FSWs’ psychological distress would be reduced upon completion of the programme and such improvements would be continued at least in the 3-month post-intervention period. The study will further explore the operation of the resilience model by specifying the association of stress perceived by FSWs, and the effects of the resilience attributes (self-esteem, self-efficacy, and coping strategies) on their level of psychological health and HIV preventive behaviours (Figure 1).

## Methods & design

### Study design

This is a randomised controlled trial in which participants will be randomly assigned to either intervention group or usual care as the control group. Participants in the intervention condition will receive the 6-session PREP, whereas participants in the control group will receive usual care. Outcome measures of all participants will be collected at baseline, completion of intervention or 6 to 12 weeks after baseline for control group, and 3 months thereafter.

### Setting

The study will take place in Hong Kong, a city in southern China. The Hong Kong AIDS Council estimated 200,000 FSWs involved in the sex industry in Hong Kong [42]. Two local non-governmental organisations (NGOs), Action for REACH OUT and JJJ Association with long-term relationship with FSWs in Hong Kong will help recruit participants and the intervention will be conducted in their service centres. AFRO was founded in 1993 and has provided extensive outreach and personal connection network with the local and migrant FSWs. AFRO’s primary target groups are mainly street sex workers, nightclub and karaoke bar girls as well as one-woman brothel workers whereas JJJ Association was set up in 2008 by a group of FSWs working in one-woman apartments, sauna and massage parlours. They provide outreach visits, assistance for family and legal problems, as well as social activities for FSWs. One of the co-investigators (WW) has worked with these organisations for many years and the long-existing collaboration will allow the present study to recruit FSWs spread across many institutions and districts in Hong Kong.

### Participants

FSWs who fulfil the inclusion criteria will be invited and have the study explained to them by the trained outreach staff of the NGOs. Inclusion criteria for the proposed study are: (i) 18 years or older; (ii) acknowledged to have been living and working as a FSW in Hong Kong within the preceding 6 months; and (iii) able to give an informed consent. All potential participants will be screened over the telephone by one of the researchers who is a clinical psychologist to confirm their fulfilment of inclusion criteria and exclude them if they are (i) unable to speak Cantonese/ Putonghua; and (ii) diagnosed with and currently on medical or psychological treatment for a serious psychological health problems such as psychosis, current substance abuse, bipolar disorder, severe affective disorders or have suicidal ideation.

Eligible participants will be provided with further details of the study and those who provide verbal consent will complete the self-reported baseline questionnaire face-to-face. Participants will be randomly allocated on a 1:1 ratio into one of the two groups according to a block randomisation list which was computer-generated. Participants are informed that they would have an equal chance of allocating into any of the two groups and the allocation outcome is unknown to participants until baseline assessment session. The women recruited will be advised not to disclose the content and activities of the intervention to others to avoid contamination. An independent researcher will conduct the baseline, post-intervention, and follow-up assessments and enter the data obtained from the questionnaires.

### Sample size

Sample size estimation is based on the “always” use condom rate during commercial transactions in the last month at 49.3% from the latest local study published on the topic [16] and an assumption of effect size of 25% on condom use rate between the intervention and control groups from a previous study [43]. In order to detect such a difference with a power of 80% and a two-tailed significance of 0.05, the sample size required is 66 in each group.

### Intervention procedure

Participants in the intervention group will receive 6 one-hour group sessions at one-to-three week intervals. There will be 3 to 6 participants in each group. These sessions target at the three major psychological resilient factors of self-esteem, self-efficacy (e.g. praise and self-rewards will be used) and coping flexibility based on resilience framework. The programme will be conducted in a group setting so that role-plays and group modelling and interactions will be used. Such an approach, informed by Social Cognitive Theory [34], espouses that individuals can learn from the actions of another person and from the regular reinforcement a person receives. Activities in these sessions will incorporate examples of their experience in daily life and at work aimed at promoting their awareness and expression of their emotions and thoughts, management of their emotions. Since the current intervention aims to promote psychological well-being, it also draws on the approach used by previous resilience interventions, including recognising and sharing positive thoughts and emotions, identifying personal strengths and ability, introducing a variety of coping strategies, and planning using their skills and strengths to achieve goals in the future. The outline of each session is listed in Table 1.

**Table 1:** Programme Outline of PREP

**Session**	**Contents**
1. Starting out	Goal-setting, encouraging awareness and expression of emotions in reaction to events
2. Empowering interpretation	Emotion management, identifying negative thoughts and generating helpful thoughts
3. Effective problem-solving	Exploring effective coping strategies, practicing problem-solving with case scenarios
4. Who am I?	Recognising their roles, giving each other affirmation
5. The unique self	Identifying successful experiences and their strengths, expressing gratitude to others
6. Preparing for the future	Exploring obstacles and using their resources to deal future challenges

The programme will be conducted by the first author, who is a clinical psychologist supervised by the investigators based on a programme manual with objectives, outlines, activities and hand-outs. While participants must attend the first and the last sessions in order, they are able to attend the other four “stand alone” session in an order according to their schedule. Based on our previous work with FSWs, special arrangements are proposed to address the practical and psychological needs to increase participation rates: (1) FSWs will be initially contacted and subsequently followed up by the outreach staff of the NGOs, whom they have established long-term contact and trust; (2) stand-alone sessions and a more flexible time intervals are unique from usual cognitive behavioural therapy schedule in order to accommodate the mobile and erratic job nature of FSWs; (3) a relatively small group size of 3–6 persons will allow the participants to share their emotions and experiences in application of the newly acquired skills.

### Usual care

Participants who are assigned to the control group will receive the standard service provision currently provided by the NGOs. Currently, the two local NGOs offer regular outreach visits and voluntary HIV/ STIs screening. Some FSWs also choose to join activities organised by the NGOs (e.g. yoga, English class or gathering). For this study, the usual service provision includes outreach visits made by the NGOs (usually the same venue will be visited once to twice a month), attending HIV/STI screening, and social activities according to their own needs and schedule. The nature of these contacts initiated by the outreach workers or sex worker themselves will be recorded. They will be advised not to join any additional psychological programmes during the study period or they have to leave the trial.

### Measures

This study will be evaluated based on a complex intervention evaluation framework . According to the guideline on developing and evaluating complex interventions published by the Medical Research Council [44], this evaluation will involve three dimensions, including assessing the effectiveness of the programme, evaluating process and assessing cost-effectiveness. As for the effectiveness of the programme, the assessment will mainly include questionnaires eliciting socio-demographic information, psychological functioning, sexual health behaviours, and programme feedback (detailed below). The questionnaires will be self-administered, with an independent rater present to clarify or assist the completion if requested.

### Effectiveness assessment - primary outcome measures

#### HIV preventive behaviour

Self-reported rate of always using a condom during vaginal sexual intercourse with clients in the previous month and at the last encounter will be used as proxy measures of safe sex behaviour and HIV prevention.

### Secondary outcome measures

#### Connor-Davidson Resilience Scale-10 items

This 10-item version of the scale [45-47] will be used to assess resilience. A higher score indicates higher resilience. The original scale shows good internal reliability and the Chinese 10-item version has shown to have good internal consistency (alpha = 0.91) and high test-retest reliability (r = .90).

#### Rosenberg self-esteem scale

It is a 10-item measure to assess global self-esteem [48] of whether participants will agree or disagree with the statements in the past 1 month on a 4-point Likert scale ranging from 1(strongly agree) to 4 (strongly disagree). Higher scores on the scale indicate higher self-esteem. One example: ‘On the whole I am satisfied with myself’. The scale has been shown to demonstrate good internal consistency and validity. The Chinese version has been shown to possess good internal consistency with alpha of 0.71.

#### Generalized self-efficacy scale

This scale contains 10 items to assess the strength of individual’s belief in their own abilities to respond to difficult situation [49,50]. Items are rated on a 4-point Likert scale, with a high total score indicating a high level of general self-efficacy. Sample item includes “I can always manage to solve difficult problems if I try hard enough”. The internal consistency (alpha = 0.86) and test-retest reliability (r = 0.75) of the original scale have been shown to be satisfactory (alpha = 0.86). The Chinese version of the scale used in the present study has demonstrated satisfactory internal consistency (alpha = 0.86).

#### Brief coping orientations to problem experienced scale (brief COPE)

Brief COPE is a 28-item questionnaire to assess behavioural and cognitive coping strategies on a 4-point Likert scale ranging from 1 (not at all) to 4 (a lot) [51]. It identifies 14 coping categories, including active coping, planning, positive reframing, acceptance, humour, religion, emotional support, instrumental support, self-distraction, denial, venting, substance use, behavioural disengagement, and self-blame. Examples of items are “I’ve been looking for something good in what is happening”, and “I’ve been blaming myself for things that happened”. The scale has been used in previous research with Chinese population [52,53]. The reliability coefficient of the instrument was 0.82 in a study with Chinese cancer patients [54].

#### General health questionnaire– 12 items (GHQ-12)

The 12-item General Health Questionnaire is used to assess the severity of non-psychotic psychiatric disturbance during the last month [55]. Sample items include ‘Been feeling unhappy and depressed’ and respondents indicate on a 4-point scale (1 = less so than usual; 4 = much more than usual) when a higher global distress score implies a higher level of psychological disturbance. The Chinese version yields satisfactory internal reliability, with alpha values ranging from 0.87 to 0.93.

#### Perceived stress scale – 4 items (PSS-4)

PSS-4 [56] contains 4-item to measure overall stress perceived by participants in the past one month. They indicate whether their lives are unpredictable, uncontrollable or overloaded on a 5-point Likert scale ranging from 0(never) to 4 (very often). A sample item includes “in the last month, how often have you felt that you felt that things were going your way”. The psychometric properties of Chinese version have been shown to be satisfactory with alpha value 0.67 and concurrent validity.

#### Risk and sexual risk behaviours

Other information on risk behaviours collected includes sexual acts, number of partners, other risk behaviours such as smoking, drug /alcohol use and dependence, health seeking behaviour, and HIV/ STI testing will also be elicited in the questionnaire.

#### Socio-Demographic information

Information to be collected includes participants’ age, years of formal education, marital status, living arrangements, monthly income and length of time working in sex industry.

### Process evaluation

The process evaluation will examine the fidelity of intervention on five different domains proposed by the National Institutes of Health Behavior Change Consortium (BCC) [57], namely design of study, training provider, treatment delivery, receipt of treatment, and enactment of treatment (see Table 2). If the programme is found to be effective, a cost-effectiveness evaluation will then be conducted.

**Table 2:** Process evaluation of PREP

**Areas**	**Goals**	**Strategies**
*Design of study*	1. To ensure intervention is developed based on relevant theory.	Literature review was conducted; intervention is based on resilience framework.
	2. To ensure “dose” across is adequately described.	Participants in intervention arm will received 6 one-hour sessions and each session one-to-three week apart; field note of each session and service received by the participants will be recorded.
*Training of provider*	3. To ensure group facilitator acquires the skills and sustains over time.	Use standardized training manuals and materials for training; role playing the sessions; external quality assurance through bi-weekly meeting with investigative team throughout the study.
*Delivery of treatment*	4. To ensure intervention delivered adheres to treatment protocol and minimize differences within intervention.	Use of an intervention manual; facilitator will take field notes of the session; Helpers will rate the adherence with a checklist; have member of investigative team to observe selected sessions; record any protocol deviations.
	5. To minimise contamination between intervention and control conditions.	Advise participants not to disclose the condition they are in; conduct interviews with selected participants in control group to ensure they do not receive treatment; train the staff of NGOs the rationale of the two conditions.
*Receipt of treatment*	6. To ensure participants understand the information provided in the sessions and they are able to perform the skills learnt.	Use of active questioning to assess their understanding; record their attendance; rate their involvement in each session by group facilitator, participants and/or session helper; Use discussion role-play to ensure participants’ understand the contents.
*Enactment of treatment*	7. To ensure participants utilise the skills introduced in the intervention sessions.	Use of homework to encourage they use their skills between sessions; discuss how they have used the skills in daily life in sessions.

An intervention manual will be used and the adherence to the manual will be constantly monitored by facilitator and investigative team throughout the study. In addition, the group facilitator will also write field notes at all stages of the research process. At the same time, the involvement of the participants on their understanding and use of the skills learnt in the sessions will be rated by the group facilitator. At post-intervention, the participants will be invited to provide feedback on their satisfaction of the programme with a Likert scale and some open-ended questions. A bi-monthly meeting will be held by the investigative team and representatives of the NGOs and sex workers for progress monitoring and program feedback. Any adverse effects of the study will be reported to the NGOs where details can be recorded and reported and discussed at the meetings. These assessments will provide information on the intervention delivery and receipt process, and allow ecological validity to the data.

### Data analyses

First of all, the baseline demographics of the intervention and control groups will be compared with independent sample *t*-tests for continuous variables and *chi*-square for categorical variables to assess the compatibility of the two-trial arms at baseline to ensure both groups were similar to start with. The change of scores on sexual health risks and psychological outcome measures (self-esteem, self-efficacy, coping, resilience and GHQ) over time between two groups will be analysed using analysis of variance with intention-to-treat analysis. Bivariate correlation analyses will be performed to examine the relationship between psychological variables and behavioural outcomes. Linear regressions will also be used to examine the associations and the effects of resilience, self-esteem, self-efficacy, coping on psychological health outcomes and HIV preventive behaviours. The statistical analyses will use SPSS 20.0 and *p*-values of 0.05 or less will be considered statistically significant.

The second objective of the current study is to explore the mechanism of how individual resilience factors influence the psychological outcomes and HIV preventive behaviours in FSWs (as proposed in Figure 1). Structural equation modelling (SEM) identifies connection strengths that best predict the observed structure of the data with respect to the specified model. Thus, SEM will be performed to evaluate the model fit of the proposed model that links psychological resources with harm-reduction behaviours as measured by condom use.

### Ethical consideration

The study protocol has been approved by the University of Hong Kong and Hong Kong West Cluster Ethics Committee (UW 12–220).

## Discussion

Previously little work has been done to evaluate the effectiveness of resilience training with a marginalised group of women internationally and in particular in the Asian region his paper describes the background and methods of a randomised controlled trial of PREP, a resilience-promoting programme, which will provide evidence for this psychological intervention for improving psychological well-being and ultimately HIV prevention. Under the framework of resilience, the intervention that focuses on individual strengths can potentially expand the understanding and its application to health promotion research by integrating the missing link between psychological and behavioural risk factors.

Current HIV prevention strategies for FSWs focus on harm reduction, biomedical and structural interventions which have operated at individual and population levels. Locally, a series of studies conducted came to a similar conclusion that HIV prevention would not be effective if boarder issues such as psychological or contextual ones were not addressed [16,58]. For example, one of our studies found vaginal douching which carried an increased infection risk, remained a common practice amongst FSWs because it helped to suppress their negative feelings of “being dirty” [59], despite extensive formal education and training to the contrary. It has become apparent to the investigative team that interventions that target fundamental issues of inequity and empower FSWs in resilience strategies are the *only* effective means at reducing the prevalence of HIV/ STIs [58]. As a contribution to scholarly knowledge, this study will contribute to the understanding of resilience among these women. Furthermore, the proposed study will enable us to evaluate the impact of the intended resilience programme on each resilience attribute, namely self-esteem, self-efficacy, and coping as mechanisms of change and whether the model of resilience could improve psychological well-being and its relation to sexual health practices among FSWs in Hong Kong.

At the individual level, a new prevention programme that addresses the psychological strength of FSWs is likely to improve psychological and physical well-being. At the societal level, it is believed FSWs with a more enhanced self-esteem may be more willing to take good care of themselves and engage in a variety of sexual health-promoting behaviours (e.g. consistent condom use), which in turn can reduce the number of HIV infection and transmission of such to their clients or partners in the general population. If successful, this study can set the foundation for future guidelines for health professionals and policymakers when considering HIV prevention strategies for other at-risk populations and managing the health needs of this sub-population and possibly other at-risk population. Further cost-effectiveness evaluation of this intervention can be conducted when the current programme is shown to be effective in improving participants’ psychological outcomes and promoting sexual health behaviors. Such a programme can be disseminated to other researchers and healthcare providers at local and international conferences as well as public education programme to improve the services provided for high-risk women.

### Limitations

Participants who volunteer for the study are arguably biased towards being more motivated and health-conscious but the design of randomised control trial equalises this effect in both groups. Furthermore there is the potential for the participants to have psychological distress and therefore screening by a clinical psychologist will help exclude those who are clinically depressed and need intensive treatment. Although the outreach staff will contact the FSW recruited regularly, the attrition rate of those who are motivated but assigned to the usual care group may be diminished and hence a potential tendency to drop out from the study. The aims and objectives of the study will be clearly explained to the potential participants and the “buy-in” will be sought before they agree to participate. Given Hong Kong is effectively one large metropolis and there are considerable overlaps between the two targeted NGOs, therefore, cluster randomised control trial will not be feasible. Even though the participants recruited from the two NGOs would be told specifically not to disclose, there may still be a risk of contamination between the intervention and control group given the participants may know each other.

## Abbreviations

AFRO: Action for REACH OUT; FSWs: Female sex workers; NGOs: Non-governmental organisations; PREP: Personal resilience and enrichment programme; STIs: Sexually transmitted infections; SEM: Structural equation modelling; WHO: World health organization.

## Competing interests

The authors declare that they have no competing interests.

## Authors’ contributions

All authors contribute to the conceptualisation of the design of the study. WW leads the overall research project. WY participated in designing and conducting the intervention program. CT, EH, AT, and WC participated in the design of the study and DF contributed to the analysis plan and interpretation. WY prepared this manuscript and all authors commented and approved the manuscript.

## Pre-publication history

The pre-publication history for this paper can be accessed here:

http://www.biomedcentral.com/1471-2458/13/683/prepub
